# Long-term outcomes of ultrasonic scalpel treatment in giant cell tumor of long bones

**DOI:** 10.3892/ol.2014.2092

**Published:** 2014-04-25

**Authors:** SHENG SUN, QIANG ZHANG, CHANG-SONG ZHAO, JUAN CAI

**Affiliations:** Department of Orthopedics, Beijing Ditan Hospital, Capital Medical University, Beijing 100015, P.R. China

**Keywords:** giant cell tumor, ultrasonic scalpel, curettage, surgical treatment

## Abstract

Giant cell tumors (GCTs) are generally benign, locally aggressive lesions with the potential to metastasize and a tendency of local recurrence. The present study aimed to investigate the advantages and long-term outcomes of application of ultrasonic scalpel in the treatment of GCT of long bones. This study retrospectively analyzed 32 cases of GCT of long bones, including 24 males and eight females. The age range was from 8 to 34 years old (mean age, 23.5 years old). The 32 cases were randomly divided into an observation group (n=10) and a control group (n=22). Patients in the observation group received curettage by ultrasonic scalpel combined with local methotrexate gelfoam adjuvant treatment, and then the cavity was filled with allograft and/or homograft bone. Patients in the control group eceived curettage by local methotrexate gelfoam adjuvant treatment and bone grafting. No local recurrence or pulmonary metastases were observed among patients in the observation group, however, six patients in the control group exhibited recurrence following surgery, although none of the patients demonstrated distant metastasis (P<0.05). Additionally, all 10 patients showed good bone knitting and rehabilitation without deformity and functional issues. The segmental bone graft was perfectly incorporated without obvious immune rejection, collapse and fracture. Curettage by ultrasonic scalpel with local methotrexate gelfoam adjuvant treatment and filling the site by allograft and/or homograft bone showed satisfactory results.

## Introduction

Giant cell tumor (GCT) of bone is locally aggressive and generally occurs in the meta-epiphyseal region of long bones. In the USA, GCT accounts for ~20% of all primary bone lesions, with a similar occurence in Asia (1–3). The presenting symptom of GCT is pain accompanied by deformity, swelling and limited joint function at the affected extremity. Occasionally, symptoms from nerve compression and pathological fracture are also identified. Shi *et al* (4) reported that the 5-, 10- and 15-year survival rates were 97, 93 and 81%, respectively, following radiotherapy treatment. The treatment of GCT is often complicated with local recurrence. Intralesional curettage is the standard of treatment for primary GCTs. Due to the high incidence of recurrence and metastasis associated with GCT, local adjuvant therapies, such as phenol or liquid nitrogen zoledronic acid, have been recommended (5–7). However, at present, there are no effective methods to prevent local recurrence and metastasis. Ultrasonic scalpels may be used to cut tissue and simultaneously avoid bleeding. Therefore, these instruments have been widely used in laparoscopic surgery. Based on the unique effect of the ultrasonic scalpel, it has been utilized to treat bone tumors (8). In the past five years, we have experienced successful treatment of GCT of long bones using this technique (9). Therefore, the present study aimed to investigate the advantages and long-term outcomes of ultrasonic scalpel in the treatment of GCT of long bones.

## Patients and methods

### Patients

This study retrospectively analyzed 32 patients with GCT of long bones, including 24 male cases and 8 female cases, who presented at the Beijing Ditan Hospital, Capital Medical University (Beijing, China) between February 2004 and February 2007. The age ranged from 8 to 34 years old (mean age, 23.5 years old), and the 32 cases of GCT were randomly divided into observation group (n=10) and control group (n=22). The 10 cases of the observation group included eight males and two females, with an age range of 8–28 years old (mean age, 22 years old). Among these 10 cases, the tumor occurrence sites were as follows: Four cases in the distal femur, two in the proximal femur, three in the proximal tibia and one in the proximal humerus. Additionally, one case with proximal femur GCT and one case with proximal humerus GCT presented with pathological fracture. The 22 cases of the control group included 16 males and six females, with an age range of 10–34 years old (mean age, 24.2 years old); The tumor occurrence sites of the control group may be broken down as follows: Eight cases in the distal femur, six cases in the proximal femur, seven cases in the proximal tibia and one case in the proximal humerus.

Plain radiographs, chest X-ray, computed tomography (CT) and/or magnetic resonance imaging (MRI) were performed on more than one plane in all patients. In addition, all patients received fine needle aspiration cytology and/or open biopsy. The thickness of the subchondral bone at the adjacent articular surface was measured, and clinical and radiographic examinations were performed regularly in the follow-up study.

The two GCT groups received intralesional curettage followed by local methotrexate treatment and bone grafting. While the observation group underwent ultrasonic scalpel for intralesional curettage.

Routine postoperative follow-up examinations were performed at 1, 3 and/or 6 months and thereafter every 6 months for 3 years. Following this, no further follow-up examination was routinely scheduled. Patients who did not experience recurrence were censored at the last follow-up study, and the mean duration of follow-up was 78 months (range, 60–96 months). Routine follow-up study included clinical examination and conventional radiography at the operative site. CT and MRI were used for further investigation when radiography demonstrated a suspected relapse (such as graft or bone resorption, expansile change and local soft tissue swelling or mass formation) or when clinical symptoms and signs showed recurrence despite negative radiography. In addition, a plain radiograph or CT of the chest was performed to exclude metastasis. Informed consent was obtained from all patients.

### Ultrasonic scalpel

The Exploiter™ ultrasonic scalpel (UOSS-II) was purchased from Beijing Beyonder Technologies Co., Ltd. (Beijing, China) and consists of three parts: The main engine, the hand shank and burr and the cooling system. The signal generator is controlled by the ultrasonic frequency electrical signal from the computer. Following amplification by the power amplifier, the electrical signal drives the ultrasonic transducer. Subsequently, the ultrasonic transducer produces a vibratory motion. The ultrasonic amplitude transformer amplifies the amplitude and drives the cutter to function. The operational frequency is 40±2 kHz. In the present study, real-time automatic frequency tracking was performed and the amplitude of the cutter was <300 μm. Additionally, 3- and 2-mm burrs were equipped with cutting teeth and notches, respectively, which were suited to the different requirements of burring. The ultrasonic energy output was set to 30% and the handle was equipped with a cooling system. Cutting tools could take the clockwise or anticlockwise and reciprocal rotation, alternately, to increase the burring ability (8).

### Surgical procedures

According to the patient’s condition, they were anesthetized by local anesthesia or general anesthesia, as appropriate. The preferred treatment of primary GCTs was intralesional curettage with high-speed ultrasonic scalpel of the tumor cavity, to improve the thoroughness of tumor removal, combined with local methotrexate gelfoam adjuvant treatment and filling of the cavity with allograft and/or homograft bone. This procedure began with sufficient fenestration as well as repeatedly scraping the inner wall of the tumor until the tumor tissue was completely invisible to the naked eye. The normal bone and epiphysial bone lamella were carefully reserved. Following this, the surgical area was rinsed repeatedly with physiological saline and then methotrexate regional chemotherapy was applied with a gelatin sponge fixed with methotrexate. For the bone transplantation, the size of the bone cavity was measured and autogenous iliac bone was harvested. If the bone cavity was too large for this, allogeneic freeze-dried bone (Osteolink Biomaterial Co., Ltd., Hubei, China) was used. One case with proximal femur GCT exhibited a pathological fracture; tumor resection and artificial total hip replacement were conducted for this patient. Furthermore, one case of proximal humerus GCT exhibited a pathological fracture, for which external fixation was employed. The control group underwent the same procedure, however rather than using the ultrasonic scalpel to scrape the inner wall of the tumor, this was undertaken using curettes.

### Statistical analysis

The Statistical Package for the Social Sciences, version 13.0 (SPSS, Inc., Chicago, IL, USA) was used for statistical calculations. All data are presented as the mean ± standard deviation. Student’s t-test was used to compare the means between the two groups, and P<0.05 was considered to indicate a statistically significant difference.

## Results

### Operation method

In total, 10 patients with GCT of the long bones received ultrasonic scalpel treatment of the tumor cavity, to improve the thoroughness of tumor removal, followed by local methotrexate gelfoam adjuvant treatment and filling of the cavity with allograft and/or homograft bone. The average bone cavity volume was 25.5 ml in observation group.

The procedure used for the observation group was successful. The time required for the procedure was shorter in the observation group (mean, 15 min) compared with that of the control group (mean, 30 min) due to the use of curettes in the control group. In the control group the field of view was unclear due to a high level of bleeding, which led to incomplete tumor removal and slight damage to the normal tissue.

### Bone healing

No rejection reaction and bone resorption phenomenon were observed in the autogenous iliac bone and allogeneic freeze-dried bone mix filling. In addition, the allograft reconstruction was successful. One case of GCT of the proximal femur received a total hip replacement, while another case of GCT in the proximal humerus received external fixation. The two cases achieved primary healing.

### Recurrence

Following surgery, tumor local recurrence and distant metastasis were not identified during the 5–8 years of follow-up among patients in the observation group; however, six cases of the control group showed recurrence following surgery, however, no distant metastasis was idetnified (P<0.05).

**Figure 1:**
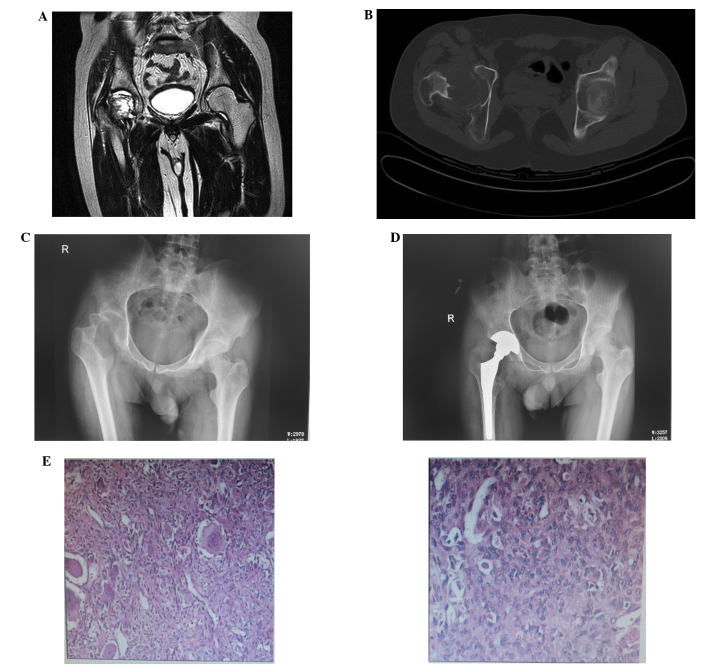
A 26-year-old male complained of pain right hip and claudication for 1 month. (A) Preoperative magnetic imaging revealing right proximal femur bone giant cell tumors. (B) Preoperative computed tomography scan showing a radiolucent, expansile, lytic lesion in the right femur head bone. (C) Preoperative radiographs showing right femur head giant cell tumors, pathological fracture and dislocation of the hip. (D) Five years following tumor resection using the ultrasonic scalpel, X-ray imaging indicates that total hip replacement and prosthesis position is good. (E) Pathological examination of resected tissue indicates cystic and necrotic tissue.

**Figure 2:**
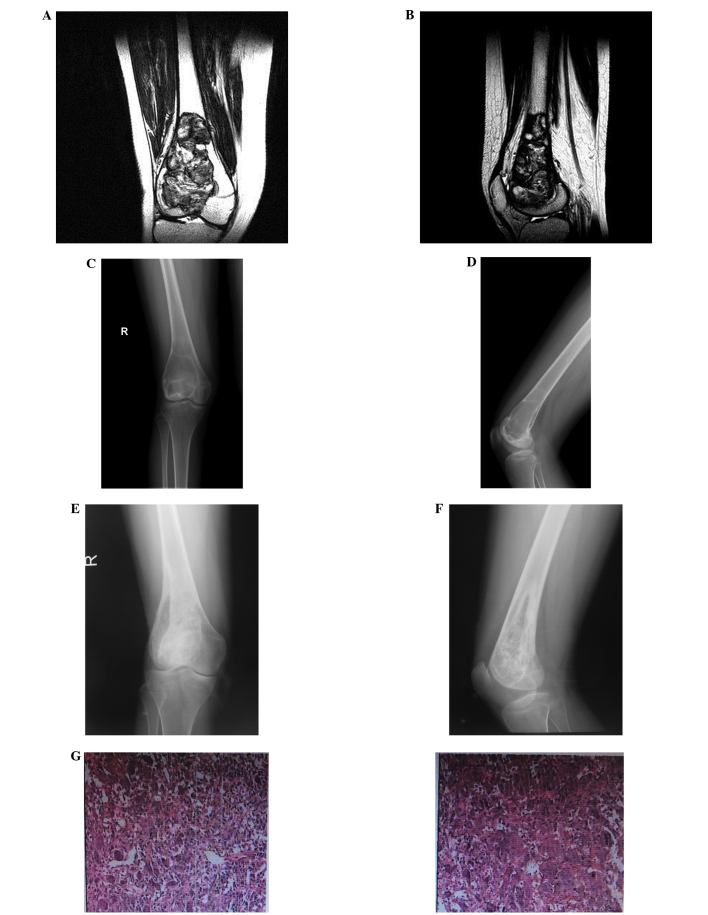
A 19-year-old female complained of pain and swelling of the right knee and claudication for 2 months. (A and B) Preoperative magnetic resonance imaging revealing right distal femur bone giant cell tumors. (C and D) GCT in the right distal femur exhibited central and multilocular growth, large erosion extent and thin cortical bone. (E) Anterioposterior and (F) five years later, lateral radiographs radiographs, showed no local recurrence of the distal femur bone giant cell tumor following ultrasonic scalpel burr curettage combined with local methotrexate gelfoam adjuvant treatment and allograft and/or homograft bone filling. No local recurrences were identified and the bone was filled successfully and healed well. In addition, no collapse or fracture of the femoral condyles was identified after six years of follow-up. (G) Pathological examination of the resected tissue indicated cystic and necrotic tissue.

All 10 cases in the observation group demonstrated good bone repair and no physical deformities, partial collapse, fracture, obvious functional issues or rejection were observed (Figs. 1 and 2).

## Discussion

Giant cell tumor (GCT) of bone is a rare benign tumor that predominantly occurs in the meta-epiphyseal region of the long bones. GCT results in disability and may be associated with a relatively high local recurrence rate (10). Chemicals (phenol and alcohol) and thermal procedures (cryotherapy and bone cement filling) have been used as adjuvants to eliminate tumor remnants. Surgical treatment options for GCT include intralesional curettage and segmental resection (7). The rate of recurrence following wide resection of bone GCTs is 6.25% (11). The overall recurrence rate of intralesional curettage was 32%. Implantation of polymethylmethacrylate instead of bone grafting has been demonstrated to be associated with a lower risk of subsequent recurrence in intralesional procedures (14 versus 50%; age range between 18.5 and 40 years) (7). However, it is not suitable for younger patients (<18.5 years old). Curettage combined with adjuvant treatment has been shown to reduce the recurrence rate to ~10%. At present, local adjuvant treatment including hyperthermia (microwave or electricity), cryotherapy (liquid nitrogen), chemical reagent daub or soaking (phenol, liquid nitrogen, carbolic acid, alcohol, 50% zinc chloride, hydrogen peroxide or zoledronic acid), high-speed abrasive drilling and pulse-rinsing can clean the tumor tissue well (12–18). The ultrasonic scalpel has developed rapidly in recent years, and owing to its selective fragmentation, low injury rate, high accuracy and the unique advantage of avoiding bleeding, it has been applied in orthopedics (9,19).

The functions of the ultrasonic scalpel in the human body include heating and cavitation, mechanical, thixotropic, dispersion, fragmentation and hemostatic effects (20,21). Three of these functions in particular, fragmentation, cavitation effect and homeostatic effect, are widely used by surgeons. Ultrasonic cutting capacity varies according to the type of tissue found in different organizational structures and their different water contents. Generally speaking, for hard or fibrous tissue, the ultrasonic burring function mainly exerts a fracturing effect, whereas for soft tissue or tissues with a high water content, it mainly exerts a cavitation effect.

During the process of fragmentation, the ultrasound propagating to the tissue causes elastic vibration (22). When the vibration acceleration reaches the cutting threshold of 50,000 × g, the biological tissue is broken due to the sharp vibration and is stripped from the surrounding tissue. The cutting threshold of 20 KHz must be reached prior to using the scalpel, and the amplitude must be >40 μm. Fragmentation plays a leading role in surgical procedures such as craniotomy and spinal decompression.

In soft tissue, such as brain and liver tumors, which has a higher water content, a large amount of bubbles are produced by ultrasound. The inner and outer pressure difference of these bubbles can reach several kilobars (1 bar=106 dyne/cm^2^). When these bubbles burst, the tissue is emulsified, which is known as the cavitation effect. The cavitation effect is closely associated with water content, and so the effect is tissue-selective (23). Owing to this feature, peripheral nerves and blood vessels cannot be incidentally damaged whilst cutting tissues such as liver and brain tumors (24,25). This feature of the ultrasonic scalpel renders it superior to other surgical instruments in use.

The present retrospective analysis indicates that the most efficient way to avoid multiple recurrences of GCT of long bone is by ultrasonic scalpel treatment of the tumor cavity, combined with local methotrexate gelfoam adjuvant treatment and filling with allograft and/or homograft bone. Thus, this procedure may be a suitable choice to minimize the risk of multiple recurrences and pulmonary metastases.

The current study identified that the ultrasonic scalpel can reduce the difficulty of the surgical procedure and shorten the operating time. The effect of burring and damaging the tumor tissue was more effective, and the ultrasonic scalpel makes the surgery safer. The working temperature of the scalpel is 70–80°C, which is sufficient to destroy the tumor cells (26,27). In addition, the surface of the wound and the bone graft were found to heal at a normal rate in the current study. When the ultrasonic scalpel is in operation, its working temperature can promote the solidification of hemoglobin, rendering simultaneous homeostasis. Compared with electric cutting and coagulation, there is less smoke, an absence of eschars and a clearer surgical field. The ultrasonic scalpel has a unique property, which is that the separation, hemostasis and cutting can work together in one machine (28,29). The device can damage and remove the tumor more completely than intralesional curettage without any damage of the normal tissue. Ultrasonic scalpel has a good application prospect due to its safety, easy control and good application effect (30).

We think that the advantages of using ultrasonic scalpel in the treatment of GCT were mainly due to its fragmentation and cavitation effects. These two functions can thoroughly clean the tumor cavity tissue even in the depth of normal bone, completely remove the source of the tumor and create a good bone graft bed. In the present patient cohort, the bone healed rapidly and there was no tumor recurrence or metastasis. Additionally, ultrasonic scalpel avoids the disadvantages of traditional treatment methods, including the fact that the tumor tissue cannot be removed thoroughly, the normal bone can undergo necrosis and the normal bone healing is delayed. The 10 cases treated with ultrasonic scalpel in the present 5- to 8-year follow-up study had no recurrence, which was an improved outcome compared with that of traditional surgery. As the sample size was small and the follow-up time was short, further study is required to determine the clinical significance of the present study findings.
